# Meeting the community halfway to reduce maternal deaths? Evidence from a community-based maternal death review in Uttar Pradesh, India

**DOI:** 10.9745/GHSP-D-12-00049

**Published:** 2013-03-21

**Authors:** Sunil Saksena Raj, Deborah Maine, Pratap Kumar Sahoo, Suneedh Manthri, Kavita Chauhan

**Affiliations:** a Public Health Foundation of India, New Delhi, India; b Boston University, Boston, MA, USA; c Indian Institute of Public Health – Delhi, Haryana, India

## Abstract

**Background:**

Uttar Pradesh (UP) is the most populous state in India with the second highest reported maternal mortality ratio in the country. In an effort to analyze the reasons for maternal deaths and implement appropriate interventions, the Government of India introduced Maternal Death Review guidelines in 2010.

**Methods:**

We assessed causes of and factors leading to maternal deaths in Unnao District, UP, through 2 methods. First, we conducted a facility gap assessment in 15 of the 16 block-level and district health facilities to collect information on the performance of the facilities in terms of treating obstetric complications. Second, teams of trained physicians conducted community-based maternal death reviews (verbal autopsies) in a sample of maternal deaths occurring between June 1, 2009, and May 31, 2010.

**Results:**

Of the 248 maternal deaths that would be expected in this district in a year, we identified 153 (62%) through community workers and conducted verbal autopsies with families of 57 of them. Verbal autopsies indicated that 23% and 30% of these maternal deaths occurred at home and on the way to a health facility, respectively. Most of the women who died had been taken to at least 2 health facilities. The facility assessment revealed that only the district hospital met the recommended criteria for either basic or comprehensive emergency obstetric and neonatal care.

**Conclusions:**

Life-saving treatment of obstetric complications was not offered at the appropriate level of government facilities in a representative district in UP, and an inadequate referral system provided fatal delays. Expensive transportation costs to get pregnant women to a functioning medical facility also contributed to maternal death. The maternal death review, coupled with the facility gap assessment, is a useful tool to address the adequacy of emergency obstetric and neonatal care services to prevent further maternal deaths.

## Abstract (precis)

Even in the face of vigorous commitment to improving maternal health services in India, inadequate staffing, supplies, and equipment at health facilities, as well as transportation costs and delays in referral, appear to contribute to a substantial proportion of maternal deaths in a representative district in Uttar Pradesh.

## INTRODUCTION

Despite progress in recent decades, India has the largest number of maternal deaths of any country in the world.^1^ Most maternal deaths in India are concentrated in 7 states (Assam, Jharkhand, Madhya Pradesh, Orissa, Rajasthan, Uttarakhand, and Uttar Pradesh). Uttar Pradesh (UP) has the second highest maternal mortality ratio (MMR) among the 7 states, at 359 maternal deaths per 100,000 live births, compared with the national average of 212 maternal deaths per 100,000 live births.^2^

The Government of India has placed special emphasis on improving maternal, newborn, and child health (MNCH) through policies and program guidelines.^3^ One major policy initiative includes increasing institutionalization of deliveries facilitated through the Janani Suraksha Yojana (JSY) program—a national conditional cash transfer scheme started in 2005 that provides eligible women with cash incentives for giving births in an institution.^4-6^

Recently, the government issued national guidelines for states to carry out maternal death reviews at both community and facility levels.^7^ However, implementation on the ground has been extremely slow and challenging.^8^ Thus, expected results have not been achieved, and the country is still at a distance from achieving Millennium Development Goal 5 (MDG 5)—reducing India's MMR to 109 by 2015.^2^^,^^9^

The maternal death review (MDR) is a tool used in many countries to understand the underlying factors leading to maternal deaths, providing programs with information to improve services and reduce MMR.^10^^,^^11^ In an effort to analyze the reasons for maternal deaths for appropriate local intervention, the Government of India introduced Maternal Death Review guidelines in 2010,^7^ based on the experience of implementing such reviews in Kerala, Tamil Nadu, and West Bengal.^12^ However, implementation has not been initiated in most of the districts.^8^

The maternal death review is a tool to help programs improve maternal services by understanding the underlying factors leading to maternal deaths.

This study was conducted to highlight key operational issues in maternal death identification in Unnao District in UP and to provide an in-depth understanding of the factors and chain of events leading to maternal deaths in the community. This information can be used to advocate policies that would enable the Government of UP to take corrective measures. The relevance of this study extends to other states in India where a similar MDR process is underway.

## METHODS

We conducted a health facility gap assessment in 15 of 16 district and block health facilities in Unnao District in UP using an instrument developed specifically for this study.^13^ Facility standards for basic emergency obstetric and neonatal care (BEmONC) and comprehensive emergency obstetric and neonatal care (CEmONC) are specified in the 2012 Indian Public Health Standards^14^^,^^15^ (see Box). We also asked community workers to identify maternal deaths occurring in the district between June 1, 2009, and May 31, 2010, and conducted verbal autopsies with families of a randomly selected subsample of the identified maternal deaths to capture factors and processes leading to the deaths.

**Box. Basic Emergency and Neonatal Care (BEmONC) and Comprehensive Emergency Obstetric and Neonatal Care (CEmONC): Standard Service Requirements in India**

According to the revised 2012 Indian Public Health Standards (IPHS), BEmONC services should be provided free of cost by Primary Health Centres and Community Health Centres while CEmONC services should be provided free of cost by District Hospitals and Community Health Centres that are designated as First Referral Units. The 2012 IPHS Standards are available at: http://mohfw.nic.in/NRHM/iphs.htm

**Table:** 

BEmONC Services	CEmONC Services
**Antenatal Care**	
Registration (within first trimester) Physical examination (weight, blood pressure, abdominal examination) Ensuring consumption of iron-folic acid (IFA) tablets (100 IFA for all pregnant women or 200 IFA for pregnant women with anemia) Essential lab investigations (Hb%, pregnancy test, urine for albumin/sugar) including blood grouping and pH typing, wet mount Assured referral linkages for complicated pregnancies and deliveries Management and provision of all emergency obstetric and newborn care for complications other than those requiring blood transfusion or surgery Linkages with nearest Integrated Counseling and Testing Centre/Prevention of Parent-to-Child Transmission (ICTC/PPTCT) Centre for voluntary counselling and testing services	All BEmONC services plus: Blood cross matching Management of severe anemia Management of complications in pregnancy referred from BEmONC
**Intranatal Care**	
Normal delivery with use of partograph Active management of third stage of labor Identification and referral for danger signs Pre-referral management for obstetric emergencies (eclampsia, postpartum hemorrhage, shock) Assured referral linkages with higher facilities Episiotomy and suturing cervical tear Assisted vaginal deliveries (outlet forceps, vacuum) Stabilization of patients with obstetric emergencies (eclampsia, postpartum hemorrhage, sepsis, shock)	All BEmONC services plus: Round-the-clock maternal care services Management of obstructed labor Surgical interventions such as cesarean section Comprehensive management of all obstetric emergencies (pregnancy-induced hypertension/eclampsia, sepsis, postpartum hemorrhage, retained placenta, shock) In-house blood bank/blood storage center Referral linkages with higher facilities including medical colleges
**Newborn Services** Neonatal resuscitation Warmth Infection prevention Initiation of breastfeeding within an hour of birth and exclusive breastfeeding thereafter Screening for congenital anomalies Weighing of newborns Antenatal corticosteroids to the mother in case of preterm babies to prevent Respiratory Distress Syndrome (RDS) Immediate care of low birth weight (LBW) newborns (>1800 g to <2500 g)	**Newborn Services**All BEmONC services plus: Round-the-clock newborn care services Care of very LBW newborns (<1800 g)
**Postnatal Care**	
Minimum 6 hours' stay post delivery 48 hours' stay post delivery and all postnatal services for days 0 and 3 for mother and baby Counseling for feeding, nutrition, family planning, hygiene, immunization, and postnatal check-up Home visits on days 3, 7, and 42 for mother and baby Additional visits for the newborn on days 14, 21, and 28 Additional visits may be necessary for LBW and sick newborns Stabilization of mother with postnatal emergencies (postpartum hemorrhage, sepsis, shock, retained placenta) Timely referral of women with postnatal complications Referral linkages with higher facilities Timely identification of danger signs and complications and referral of mother and baby	All BEmONC services plus: Clinical management of all maternal emergencies such as postpartum hemorrhage, puerperal sepsis, eclampsia, breast abscess, postsurgical complication, shock, and any other postnatal complications such as RH incompatibility
**Newborn Services** Warmth Hygiene and cord care Identification, management, and referral of sick neonates, LBW, and preterm newborns Care of LBW newborns (<2500 g) Zero day immunization–OPV (oral polio vaccine), BCG (bacille Calmette-Guerin for tuberculosis), Hepatitis B Care of LBW newborns (>1800 g to <2500 g) Referral services for newborns <1800 g and other newborn complications Management of sepsis	**Newborn Services**All BEmONC services plus: Newborn care in district hospitals through Sick Newborn Care Unit (SNCU) Management of complications Care of very LBW newborns (<1800 g) Establish referral linkages with higher facilities

Source: Reference 14

### Study Site and Demographic Profile

We chose UP as the study state because it has the second highest MMR in India and it was one of the intervention states under the Maternal and Child Health Sustainable Technical Assistance and Research (MCH-STAR) initiative to improve maternal, neonatal, child health, and nutrition policies and programs in India. We selected Unnao as the study district because it ranked in the middle range of socioeconomic status among the districts in UP (48^th^ out of the 70 state districts), based on a composite index of 13 socioeconomic and demographic indicators.^16^

In 2010, Unnao District had a total population of about 3 million, with a birth rate of 22.2 per 1,000 people (Table 1).^17-18^ An estimated 69,055 births are reported annually and an estimated 248 maternal deaths occurred based on the MMR of 359 (308–409) in UP.^2^^,^^19^ Although recent estimates have shown a significant drop in the MMR, by 59% at the national level between 1990 to 2007, the reduction in UP was only 12%, from 407 to 358.^20^ Although these estimates are based on the Sample Registration System conducted by the government, they are the most reliable estimates currently available.

**Table 1.** Demographic Characteristics, Unnao District, Uttar Pradesh, 2010

Characteristic	Data
Total area (km^2^)	4,558
No. of blocks	16
Total population	3,110,595
Birth rate (per 1,000 people)	22.2
Estimated no. of annual births	69,055
No. of institutional deliveries^a^	14,488
Estimated no. of maternal deaths	248
No. of district hospitals	1
No. of Community Health Centres (CHCs)	4
No. of CHCs working as First Referral Units (FRUs)	2
No. of Block Primary Health Centres	9
No. of Anganwadi centres	2,376
No. of Anganwadi workers (AWWs)	2,573

a Data from the District Program Management Unit, Unnao, 2011.

Source: References ^17^^,^^18^

Unnao has 1 District Hospital (DH), 4 Community Health Centres (CHCs), of which 2 are designated as First Referral Units (FRUs), and 9 Block Primary Health Centres (BPHCs).

### Data Collection and Analysis

We asked key informants to report on all maternal deaths due to any cause in women ages 15 to 49. Key informants included auxiliary nurse midwives (ANMs) and accredited social health activists (ASHAs), who work under the Ministry of Health and Family Welfare, and Anganwadi Workers (AWWs) who work under the Ministry of Women and Child Development.

Key informants reported a total of 207 maternal deaths from all the blocks in Unnao District, excluding the 2 urban blocks of Shuklaganj and Unnao City. Of these 207 deaths, we confirmed that 153 were maternal deaths that occurred during the study period, representing 62% of the estimated 248 maternal deaths occurring in Unnao District in 2010. The AWWs reported all 153 maternal deaths, but the ANMs and ASHAs did not capture 30% of the deaths. We validated 10% of key informants' maternal death reports with families either through in-person visits or by telephone.

**FIGURE 1.**
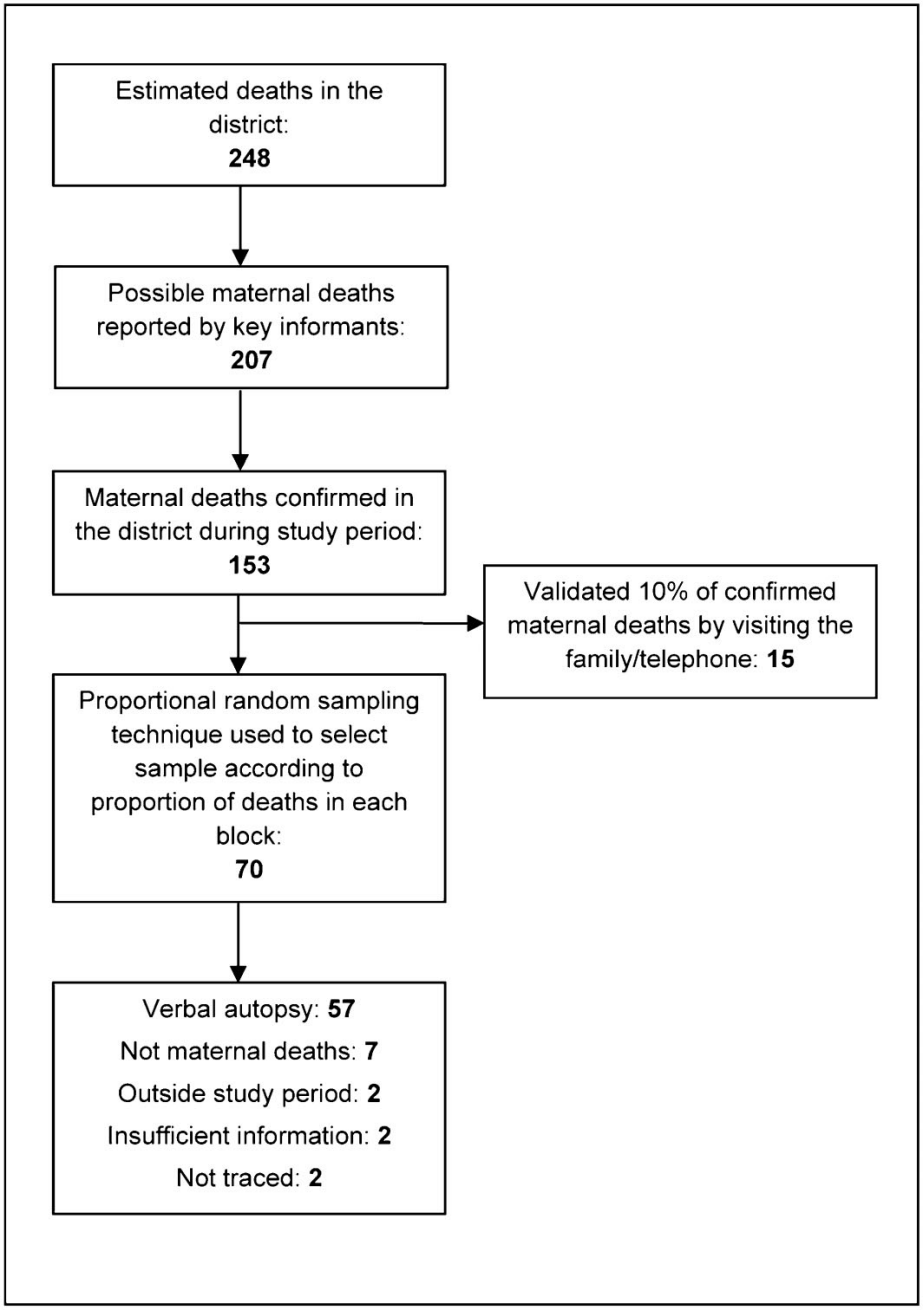
Sample Selection Strategy

Of the 153 deaths occurring during the study period, we randomly selected a sample of 70 deaths, in proportion to the deaths in each block, to conduct verbal autopsies. We used a modified Maternal and Perinatal Death Inquiry & Response tool—a detailed verbal autopsy questionnaire—to capture missing links in officially recorded data and reconstruct the sequence of events to help pinpoint the exact cause of a maternal death.^21^ We administered the questionnaire to husbands of the deceased and to women in the household for 57 of the sampled 70 cases; we subsequently found that 7 deaths were not related to pregnancy or childbirth, 2 were outside the study period, 2 cases had insufficient information, and 2 cases could not be traced (Figure 1). The recall period in all deaths was less than 1 year, as the study was conducted in the last quarter of 2010.

The completed verbal autopsy forms were reviewed by 2 obstetricians who independently assigned probable cause of death, based on available information recorded in the questionnaires related to history, events, and symptoms. If the diagnoses of the 2 obstetricians matched, we considered the diagnosis final. In cases of disagreement, a third obstetrician reviewed the form, and we considered her diagnosis final.

Approval for the study was obtained by the Institutional Ethics Committee of the Public Health Foundation of India. All interviews were conducted after obtaining written consent from the family. Data collected was coded and analyzed, and emergent themes were documented and studied to understand their implications with respect to reducing maternal mortality. All the data were analyzed anonymously using SPSS 17^©^.

## RESULTS

### Demographic Characteristics of Identified Maternal Deaths

The mean age of the women in the sample of identified maternal deaths was 27.5 years (standard deviation, 4.8) with a minimum of 18 years and maximum of 38 years. Around 54% of women and 45% of their husbands were illiterate. About 56% of the women lived in a Kutcha house (mud and straw structures), where 70% did not have a toilet or electricity in their houses. Almost half of the women were below the poverty line, 98% were Hindu, and 44% were from the disadvantaged Scheduled Caste category (Table 2).[Table t03]

**Table 2.** Demographic Characteristics of Identified Maternal Deaths, Unnao District, Uttar Pradesh (n = 57)

Characteristics	No. (%)
**Age** at the time of death, mean (standard deviation), years	27.5 (4.8)
**Women's education**	
Illiterate	31 (54.4)
Literate	13 (22.8)
Do not know	13 (22.8)
**Husband's education**	
Illiterate	26 (45.6)
Literate	29 (50.9)
Do not know	2 (3.5)
**Religion**	
Hindu	56 (98.3)
Muslim	1 (1.8)
**Caste**	
Scheduled Caste^a^	25 (43.9)
Scheduled Tribes^a^	1 (1.8)
Others	31 (54.4)
**Type of house**	
Kutcha	32 (56.1)
Kutcha-pucca	13 (22.8)
Pucca	12 (21.1)
**Toilet in the house**	
Yes	17 (29.8)
No	40 (70.2)
**Electricity in the house**	
Yes	17 (29.8)
No	40 (70.2)
**Below Poverty Line card**^b^	
Yes	27 (47.4)
No	28 (49.1)
Do not know	2 (3.5)

a “Scheduled Castes” and “Scheduled Tribes” are historically disadvantaged communities.

b Can be used to access all the welfare schemes provided by the Government of India.

### Findings From the Facility Gap Assessment

#### Basic Emergency Obstetric and Neonatal Care Facilities

All 15 of the facilities assessed should have been providing at least BEmONC services, according to government guidelines.^22^ Our findings showed that none of the facilities met the recommended standards for BEmONC except the district hospital. Two-thirds of the facilities studied did not report treating any women with maternal complications in the 3 months prior to the study period. One-third of the facilities did not have antibiotic injections available to manage infection in the labor room. None of the 15 facilities had injectable magnesium sulphate available for management of eclampsia (hypertensive disorder), and 40% did not have parenteral oxytocin in the labor room to treat postpartum hemorrhage, while 67% did not have it in store. Misoprostol was also not available in 53% of the facilities.

None of the health facilities assessed, except the district hospital, met the recommended standards for basic emergency obstetric and neonatal care.

None of the facilities reported treating any women for abortion-related complications. Although 24/7 normal delivery care is theoretically available in all 15 facilities, 24/7 assisted deliveries (vacuum extraction, forceps) were conducted in only 2 facilities. Nearly all (95%) of the assisted deliveries in the district were conducted in the district hospital.

Regarding neonatal care, 20% of the facilities did not have a designated newborn baby corner. Nearly three-quarters did not have a weighing scale and bulb syringe (to remove mucus), while 80% did not have an overhead radiant warmer. A functioning, self-inflating Ambu bag and face mask (for neonatal resuscitation) were not available in 93% of the facilities. About half of the facilities did not have a functional oxygen supply.

#### Comprehensive Emergency Obstetric and Neonatal Care Facilities

Three of the facilities (1 district hospital and the 2 FRUs) assessed should have been providing CEmONC services, according to government guidelines. Nearly all cesarean deliveries were conducted at the district hospital.

Both the designated FRUs had an obstetrician, anesthesiologist, and pediatrician in position, but round-the-clock services were not available except in the district hospital. Two BPHCs with 24/7 delivery services did not have even a female medical officer while 7 of the BPHCs did not have a qualified staff nurse (obstetrical services are mostly provided by female medical officers and staff nurses), falling short of the 2012 revised Indian Public Health Standard (IPHS) Guidelines.^15^^,^^23^ The facilities lacked essential equipment and instruments and the condition of the labor room, including the walls, flooring, ceiling, lighting, and water supply, was unsatisfactory. Similarly in the FRUs, the overall condition of the operating table (in 7 facilities), infrastructure (in 9 facilities), and cleanliness (in 7 facilities) was found to be unsatisfactory.

Furthermore, 40% of the facilities did not have a functional ambulance available, and among those with a functional ambulance, 47% did not have adequate funds to operate the ambulance. No facilities had referral transport available round-the-clock. Out of 9,355 deliveries reported by the facilities in the 3 months prior to the gap analysis, only 260 cases (less than 3%) were known to have been referred, which is much lower than the general norms of about 15% of women who will need either BEmONC or CEmONC services.^24^ Although all the facilities could conduct hemoglobin estimation, 79% of the facilities did not conduct even 2 hemoglobin investigations a day. Finally, 67% of the facilities did not have any staff trained in waste management and 20% did not have a 24-hour running water supply.

### Findings From the Verbal Autopsy

**FIGURE 2.**
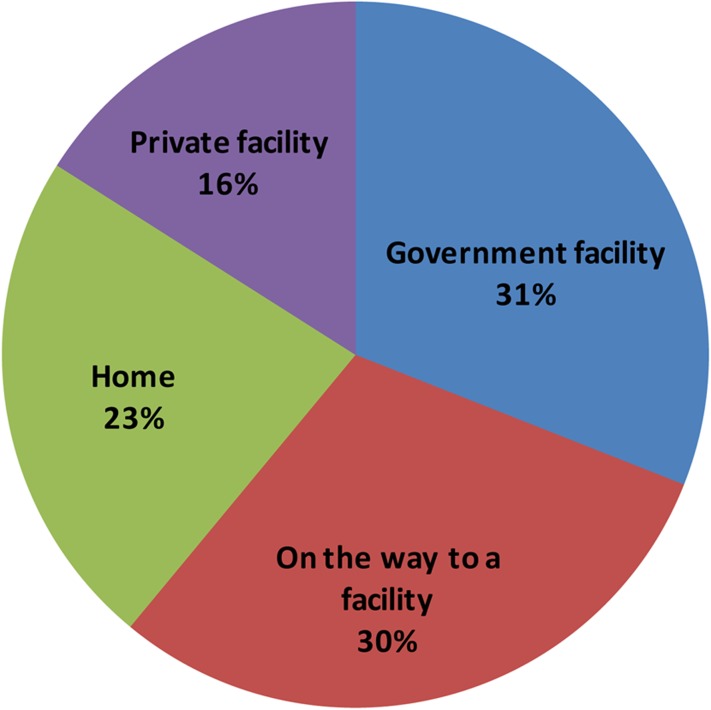
Reported Place of Death in Maternal Death Reviews, Unnao District, Uttar Pradesh, 2010–2011

Verbal autopsy revealed that 16% of the maternal deaths reportedly occurred in a private facility, 30% in a government hospital, and 23% at home, while 30% died on the way to a facility (Figure 2). Our study did not identify any abortion-related deaths, which is most likely due to underreporting by the family. Even though abortion is legal in India, many studies have shown that it is still an important cause of maternal death, due to lack of access to safe abortion services.^25^ Of the 13 maternal deaths that took place at home, 85% of these women were illiterate, 77% had illiterate husbands, and 54% belonged to the Scheduled Caste and were below the poverty line (Table 3). Of the 17 maternal deaths occurring on the way to a facility, 35% of the women and their husbands were illiterate, 41% belonged to the Scheduled Caste, and 47% were below the poverty line. Of the 27 maternal deaths that took place in the facility, about half of the women were illiterate, 41% belonged to the Scheduled Caste, and 44% were below the poverty line. Only 15 of these 27 women (56%) had sought antenatal care at least once from the facilities.

**Table 3. t03:** Reported Place of Maternal Death by Background Characteristics

Characteristics	Place of Maternal Death, No. (%)			
	Home	On the way to a facility	Facility	*P* Value^a^
**Women's education**				
Illiterate	11 (84.6)	6 (35.3)	14 (51.9)	
Literate	1 (7.7)	6 (35.3)	6 (22.2)	0.11
Do not know	1 (7.7)	5 (29.4)	7 (25.9)	
**Husband's education**				
Illiterate	10 (76.9)	6 (35.3)	10 (37.0)	
Literate	3 (23.1)	11 (64.7)	29 (55.6)	0.07
Do not know	0 (0)	0 (0)	2 (7.4)	
**Religion**				
Hindu	13 (100.0)	16 (94.1)	27 (100.0)	0.30
Muslim	0 (0)	1 (5.9)	0 (0)	
**Caste**				
Scheduled Caste^b^	7 (53.9)	7 (41.2)	11 (40.7)	
Scheduled Tribe^b^	0 (0)	0 (0)	1 (3.7)	0.79
Others	6 (46.2)	10 (58.8)	15 (55.6)	
**Below Poverty Level card**^c^				
Yes	7 (53.9)	8 (47.1)	12 (44.4)	0.65
No	6 (46.2)	9 (52.9)	13 (48.2)	
Do not know	0 (0.0)	0 (0)	2 (7.4)	
**Received Antenatal Care**				
Once	1 (25.0)	0 (0)	1 (6.7)	
Twice	1 (25.0)	4 (44.4)	6 (40.0)	0.56
Three times or more	2 (50.0)	4 (44.4)	8 (53.3)	
Do not know	0 (0)	1 (11.1)	0 (0)	
**Total**	13 (100)	17 (100)	27 (100)	

a *P* values < 0.05 were considered statistically significant.

b “Scheduled Castes” and “Scheduled Tribes” are historically disadvantaged communities.

c Can be used to access all the welfare schemes provided by the Government of India.

Verbal autopsies indicated that almost half of maternal deaths occurred at a health facility.

According to family responses (available for 28 cases), only 1 woman planned to deliver at home, yet 24 of the 57 women (Table 4) delivered at home. In terms of the timing of the deaths, 9 women died before delivery and the remaining 48 women died after delivery (Table 4). Of the 48 women dying after delivery, 25% died (12) at home, 27% (13) on the way to a facility, and 48% (23) in the facility, and all 48 women died within 6 weeks or 42 days of delivery.

**Table 4.** Cause of Maternal Death by Reported Place of Death

	Place of Maternal Death, No. (%)			
	Home	On the way to a facility	Facility	Total
Maternal deaths	13 (22.8)	17 (29.8)	27 (47.4)	57 (100.0)
Maternal deaths after delivery^a^	12 (25.0)	13 (27.2)	23 (47.9)	48 (100.0)
**Cause of Death**				
Hemorrhage	6 (46.2)	7 (41.2)	9 (33.3)	22 (38.6)
Severe anemia	4 (30.8)	5 (29.4)	6 (22.2)	15 (26.3)
Sepsis	2 (15.4)	1 (5.9)	5 (18.5)	8 (14.0)
Pregnancy-induced hypertension and eclampsia	1 (7.7)	3 (17.7)	2 (7.4)	6 (10.5)
Obstructed labor	0 (0)	1 (5.9)	3 (11.1)	4 (7.0)
Unknown	0 (0)	0 (0)	2 (7.4)	2 (3.5)
**Total**	13 (100)	17 (100)	27 (100)	57 (100)

a Of 57maternal deaths, 48 women died after delivery while 9 died during pregnancy.

Based on the verbal autopsies (and, in 2 cases, medical records available from the relatives), the major direct causes of the reported deaths were: 22 cases of hemorrhage (38%), 15 cases of anemia (26%), 8 cases of sepsis (14%), 6 cases of eclampsia (10%), 4 cases of obstructed labor (7%), and 2 cases that could not be diagnosed with available information (4%). No death certificates were available to validate the cause of death.

Table 5 shows factors contributing to the maternal deaths, which can be grouped into 3 main categories using the “3 Delays Model.”^26^

**Table 5.** Factors Causing Delays in Accessing Appropriate Maternal Health Care (n = 57)

Delay Factors	Facility 1	Facility 2	Facility 3
Sought care at and reached a facility (%)	80.7	56.1	24.6
Mean time to make arrangements/travel from the previous location to the next (hrs)	3.1	9.9	3.1
Mean travel time from the previous location to the next (hrs)	1.0	1.4	1.6
Median distance from the previous location to the next (km)	11.0	31.5	25.0
Median cost of transport from the previous location to the next (Rs)^a^	100	600	550
Median duration of stay (hrs)	2.0	3.0	3.0
Median cost of care (Rs)^a^	375	1,500	2,500
Had cash to seek care (%)	43.6	37.0	0
Borrowed money (%)	56.4	55.6	100.0
Sold assets (%)	0	3.7	0

a US$1 ≈ Rs. 55

**Delay 1 (delay in deciding to seek care):** The most common reason that families cited for not seeking care sooner was lack of available transport (28%). Another reason cited was the cost of transport. The median cost of transport from home to the first facility was Rs. 100 (US$1.83); from facility 1 to facility 2, Rs. 600 (US$10.96); and from facility 2 to facility 3, Rs. 550 (US$10.04). The mean time spent deciding to take the pregnant women to a facility was 4 hours, and average time to make arrangements was 3 hours. It is easy to see how time could be lost while families weighed all these factors.

**Delay 2 (delay in reaching adequate facility):** According to family reports, by far the most common mode of transport to all facilities was taxi/auto rickshaw/tractor. Only during transfers from the second to third facility did the families mention travel by ambulance provided by the health facility. Being sent from one facility to another (and another) adds dangerous delay. More than half the families had to borrow money to take women to the first facility, while all the families borrowed money for treatment. Although public facilities provide free care, families still had out-of-pocket expenses, such as costs for transportation and medicines (from outside pharmacy as advised by the doctor of the visited facility). The median cost of care in the first facility was Rs. 375 (US$6.85); in facility 2, Rs. 1,500 (US$27.39); and in facility 3, Rs. 2,500 (US$45.66).

Many families had to borrow money to cover costs of transportation and medical care.

**Delay 3 (delay in receiving adequate care at facility):** While attention is often focused on delays 1 and 2, information provided by the MDR clearly shows that delays in receiving appropriate care once at the facility played an important role in the maternal deaths in Unnao District. Half of the women had to be taken to at least 2 facilities for management of their complications, thus losing precious time (the mean travel time between facility 1, facility 2, and facility 3 is 1.3 hours).

Half of the deceased women were taken to at least 2 facilities, causing fatal delays in receiving appropriate care.

## DISCUSSION

A community-based maternal death review can be a useful tool for program planners, managers, and health advocates, provided that the data are used appropriately and strengths and weaknesses of the tool are kept in mind.^27^ Strengths of the MDR lie in the depth of information that can be gathered on the process that the pregnant woman and her family went through and the barriers that they faced. Limitations of the MDR are directly related to its strengths, as it cannot provide maternal mortality levels in the study area.

Although the MDR conducted in Unnao District represents only 1 district in UP, we believe it is representative of the general situation of maternal care in the state. The difficulties of identifying maternal deaths have been reported in both developed and developing countries for decades. In our study, we took advantage of the extensive network of community health workers in India (ANMs and ASHAs) to report maternal deaths that might have escaped official notice. An important finding from the death identification process is the high proportion of maternal deaths reported by Anganwadi workers, who are employed by the Ministry of Women and Child Development rather than the Ministry of Health and Family Welfare.

Anganwadi workers identified more than half of the expected maternal deaths in the district.

By comparing the expected number of deaths in the study area in a year to the number reported by the community workers, we estimate that we identified about 62% of maternal deaths. This is not an unusual finding. It seems that the only way to identify nearly all maternal deaths in developing countries is to do a repeat household survey and explore the absence of any person. This has been done for many years in Matlab, Bangladesh.^28^ Unfortunately, it is too expensive and time consuming to do this for a large area (such as an entire district in UP). Moreover, precise data on the MMR are not needed to identify and address problems in the health system, as our data show.

Clearly the ability of verbal autopsy to identify exact cause of death is limited. An unusually high level (even for India) of maternal deaths was attributed to anemia (26%), which may partly represent deaths related to hemorrhage. Our experience confirmed that of many earlier researchers who found that medical causes of many maternal deaths were not reported, even when a variety of community methods were used.^29^ We are aware that the distribution of medical causes of death is inexact in this data set, but it does provide an idea about the major causes of maternal death in the community, for which an appropriate strategy can be formulated. This is consistent with findings in other studies that found that verbal autopsies have limited validity in the attribution of maternal deaths to single specific medical causes and that multiple causes of death should be considered in determining program priorities.^29^^,^^30^

This study revealed several important findings in terms of the maternal death reporting process. One is that very few maternal deaths were reported by the government health facilities studied. A common explanation for this might be that women with complications stay at home and either die there or on the way to a facility. Our data, however, show that this is not a valid explanation, as many women are reported to have died in facilities, but the community workers—not the facilities—reported these deaths. A potential reason that facilities may underreport maternal deaths could be to avoid investigation and punitive action by higher authorities. Under the new MDR guidelines, the Government of India has clearly pronounced a “no punitive action” policy but how much this has eliminated apprehension is yet to be seen. This is clearly an area for further study.

Our study also revealed important areas of the health service delivery system that could be strengthened to reduce maternal mortality, particularly ensuring that facilities provide the appropriate level of emergency obstetric and neonatal care services and that the referral system is efficient and effective. Even First Referral Units with qualified specialists did not manage complications of pregnant women due to such factors as lack of blood or unavailability of staff, and instead transferred the women to the district hospital. In most cases, families tried desperately to obtain medical care for the women, traveling to one, then a second, and often a third, medical facility. Not only did this consume precious time and money (often borrowed), but also many women and their babies died along the way. Our data also highlight the fact that, even after recognition of a complication by health staff, women were referred to the next higher level of facility in the hierarchy—that is, from BPHC to CHC to district hospital—rather than directly to a facility where appropriate resources were available to manage the complication.

Lack of the appropriate level of care at health facilities and a poor referral system were key factors contributing to maternal deaths.

Finally, our findings indicate that a woman's family spends twice the amount in seeking care as that given by the government to pregnant women under the Janani Suraksha Yojana (JSY) scheme that aims to promote institutional deliveries. There are nearly 8.37 million JSY beneficiaries in India—about 37% of whom are in UP. In UP, institutional deliveries constitute 47%, and home deliveries 52%, of all deliveries. About 76% of women in UP were aware about the JSY scheme and 38% of the JSY beneficiaries belong to households that are below the poverty line. Among JSY beneficiaries in UP, 72% of the mothers received money (Rs. 1,400 [US$ 26.04] or more) after the institutional delivery, but only 8% of them received the amount at the time of discharge.^31^ However, our study found that families in Unnao District, UP, spent almost Rs. 500 (US$9.30) for transportation and care costs at the first facility, Rs. 2,100 (US$39.06) at the second facility, and about Rs. 3,000 (US$55.80) at the third facility.

In the case of Tamil Nadu, the government has enhanced the Muthulakshmi Reddy Maternity Benefit Scheme to Rs. 12,000 (US$223.21).^32^ While the top priority should be focused on improving the service delivery system, the Government of India may also consider increasing the JSY incentive while state governments can initiate and implement special schemes focused on maternal and child health to reduce maternal and infant mortality.

### Study Limitations

The MDR can tell us a great deal about the process leading to maternal deaths. However, it cannot tell us much about the level and medical causes of maternal deaths for comparison among sites or over time.^30^ A related limitation concerns the distribution of clinical causes of maternal death. Another weakness of the MDR is that information about reasons for delay comes from key informants such as family members, who might be less likely to attribute delays in seeking services to hesitation on the part of the family.

In our study, we know that the reported cases are not representative of all maternal deaths, because no abortion-related deaths were identified. There may also be other, less obvious biases. Furthermore, we obtained information on only one-third of identified maternal deaths. Nevertheless, the cases we followed were selected randomly from the sample of identified maternal deaths, and our findings on reasons for delay were so consistent that we believe they are representative.

## CONCLUSION

Combining data gathered during interviews with families of deceased pregnant women with information gathered during facility gap assessments provides a valuable picture of what families face when a woman develops a serious obstetric complication. Our findings indicate that the expense of transporting a pregnant woman to a functioning medical facility is one of the major contributing factors to maternal death. Life-saving treatment of obstetric complications is also generally not offered at the appropriate level of government facilities and an inadequate referral system contributes to fatal delays in receiving appropriate care. Therefore, the government needs to focus on strengthening facilities that provide emergency obstetric and neonatal care services and on developing a functional and effective referral system. Furthermore, we would like to thank USAID-India, for providing financial support to the study through the MCH-STAR initiative.
